# Is atmospheric pressure change an Independent risk factor for hemoptysis?

**DOI:** 10.12669/pjms.303.5063

**Published:** 2014

**Authors:** Omer Araz, Elif Yilmazel Ucar, Metin Akgun, Yener Aydin, Mehmet Meral, Leyla Saglam, Hasan Kaynar, Ali Metin Gorguner

**Affiliations:** 1 Dr. Omer Araz, Assistant Professor, Department of Pulmonary Diseases, Ataturk University School of Medicine, 25240, Erzurum, Turkey.; 2 Dr. Elif Yilmazel Ucar, Assistant Professor, Department of Pulmonary Diseases, Ataturk University School of Medicine, 25240, Erzurum, Turkey.; 3 Dr. Metin Akgun, Professor, Department of Pulmonary Diseases, Ataturk University School of Medicine, 25240, Erzurum, Turkey.; 4 Dr. Yener Aydin, Assistant Professor, Department of Thoracic Surgery, Ataturk University School of Medicine, 25240, Erzurum, Turkey.; 5 Dr. Mehmet Meral, Professor, Department of Pulmonary Diseases, Ataturk University School of Medicine, 25240, Erzurum, Turkey.; 6 Dr. Leyla Saglam, Professor, Ataturk University School of Medicine, 25240, Erzurum, Turkey.; 7 Dr. Hasan Kaynar, Professor, Ataturk University School of Medicine, 25240, Erzurum, Turkey.; 8 Dr. Ali Metin Gorguner, Professor, Department of Pulmonary Diseases, Ataturk University School of Medicine, 25240, Erzurum, Turkey.

**Keywords:** Atmospheric Pressure, Hemoptysis, Humidity, Outdoor Temperature

## Abstract

***Objective:*** Hemoptysis is one of the most important and challenging symptoms in pulmonary medicine. Because of the increased number of patients with hemoptysis in certain periods of the year, we aimed to investigate whether atmospheric changes have an effect on the development of hemoptysis with or without a secondary cause.

***Methods:*** The data of patients presenting with hemoptysis between January 2006 and December 2011 were analyzed. Data on the daily atmospheric pressure (hectopascal, hPa), relative humidity (%), and temperature (^o ^C) during that time were obtained.

***Results:*** A total of 232 patients with hemoptysis, 145 male (62.5%) and 87 female (37.5%) with an average age of 48.1(±17.6), were admitted to our hospital between 2006 and 2011. The highest admission rates were in the spring season, the highest in May (n=37, 15.9%), and the lowest admission rates were in December (n=10, 4.3%). A statistically significant negative correlation was found between the number of hemoptysis cases and mean atmospheric pressure but no relative humidity or outdoor temperature.

***Conclusion:*** Hemoptysis is very much influenced by weather factors; in particular, low atmospheric pressures significantly affect the development of hemoptysis. Fluctuations in atmospheric pressure may also play a role in hemoptysis.

## INTRODUCTION

Hemoptysis, or the expectoration of blood, is one of the most important and challenging symptoms in pulmonary medicine, and it can range from the blood streaking of sputum to the presence of gross blood in the absence of any accompanying sputum. True hemoptysis is usually accompanied by coughing, characterized by bright red blood that may be frothy with an alkaline pH and contains no food particles.^1^

Bronchiectasis, pulmonary tuberculosis, and lung cancer are the three main causes of hemoptysis. The frequency of each disease as a cause of hemoptysis varies according to the geographical area. Pulmonary tuberculosis is an important cause of hemoptysis in developing countries,^2^^,^^3^ whereas in developed countries, bronchiectasis, lung cancer, and bronchitis are the main causes.^4^^,^^5^ Lung cancer, bronchiectasis, and tuberculosis are the main causes of hemoptysis in Turkey.^6^^,^^7^ However, despite advances in diagnostic methods, the cause goes undetected in nearly a third of all cases of hemoptysis.^8^

Because of a seasonal increase in the cases admitted to our hospital, we considered that environmental factors might play a role in the presentation of hemoptysis that occurs in cases with pulmonary embolism or spontaneous pneumothorax. For this reason, we aimed to investigate whether atmospheric alterations (including atmospheric pressure, outdoor temperature, and humidity changes) play a role in the presentation of cases with hemoptysis, both idiopathic and secondary to various diseases. We also aimed to determine which atmospheric alteration is associated with the development of hemoptysis.

## METHODS

***Patients: ***A total of 402 patients presenting with hemoptysis between January 2006 and December 2011 were included in the study, 170 of them were removed on account of exclusion criteria. Their demographic data, clinical history, diagnosis, application dates, the date of onset of hemoptysis, and clinical findings (including computerized tomography of the thorax and bronchoscopy) were obtained from the records of the Ataturk University Research Hospital and were evaluated retrospectively.

The criteria for inclusion required that the patient was located on the immediate surroundings territory of Erzurum when hemoptysis occurred. Cases with either massive or minor hemoptysis were included in the study. We defined massive hemoptysis as either ≥ 200 mL of expectorated blood over a 24-hour period or bleeding at a rate ≥ 50 mL/hour, regardless of whether abnormal gas exchange or hemodynamic instability existed.^9^ Cases who were not diagnosed by a physician or with no hemoptysis as assessed upon bronchoscopy; were younger than 16 years old; had no CT of the thorax(n=5, because of contraindications); had no data regarding a near, nose, and throat examination; had a suspicion of gastrointestinal hemorrhage; or had no information on the exact date of onset of hemoptysis were excluded from the study.

***Meteorological Data: ***The meteorological data (including daily mean atmospheric pressure (hectopascal, hPa), mean relative humidity (%), and mean temperature (^o ^C) between 2006 and 2011 were obtained from the Turkish State Meteorological Service.^10^ The data from the day of the event and the previous two days were used in calculations. They were given as mean values (the mean of three days unless otherwise indicated).

***Statistical Analysis: ***The data were analyzed using SPSS version 18 statistical software (SPSS Inc., Chicago, IL). The Pearson correlation test was used to assess the following: number of cases and atmospheric pressure, relative humidity, and outdoor temperature on the day of hemoptysis and on the preceding three days. The chi-square test was used to evaluate massive hemoptysis and its association with the presence of a cause, bronchoscopic findings, or presented month or session. P < 0.05 was considered statistically significant.

The study was planned according to the ethics guidelines of the Helsinki Declaration, and the study protocol was approved by the Ataturk University School of Medicine local ethics committee (B.30.2.ATA.0.01.00/01).

## RESULTS

A total of 232 patients with hemoptysis, 145 male (62.5%) and 87 female (37.5%) with an average age of 48.1 (± 17.6), were admitted to our hospital between 2006 and 2011.Of these, 106 (45.7%) were smokers with a 37.2 ± 26.2 pack/year smoking history. Massive hemoptysis was seen in 73 cases (31.5%). The highest admission rate was in May (n=17). The mean atmospheric pressure in May was 821.8 hPa. There was no statistically significant difference between massive hemoptysis and months. The diagnoses of the cases are shown in Table-I. Of the cases, 20 (8.6%) were idiopathic. Bronchoscopy was performed in 115 cases (49.6%), and 34 (14.7%) had endobronchial lesions. Hemoptysis was observed in all the cases who had undergone bronchoscopy, but the origin of hemoptysis was determined in 76 of the cases (66.0%), with the most common diagnoses being bronchial cancer (10.3%), bronchiectasis (5.6%), and chronic obstructive pulmonary disease (5.5%). 

**Table-I:** The diagnosis of all patients

*Diagnosis* *	*Number of cases*	*%*
Pneumonia Bronchiectasis COPD	62 39 37	26.7 16.8 15.9
Lung Cancer Tuberculosis Hypertension	28 25 24	12.1 10.8 10.3
Cardiac Diseases	21	9.1
Vasculitis Drug^ϔ^ Hematologic Diseases	11 10 6	4.7 4.3 2.6
Pulmonary embolism	6	2.6
Carcinoid	5	2.2
CVD	2	0.9
Idiopathic Other diseases	20 17	8.6 7.1

COPD: Chronic Obstructive Pulmonary Disease, CVD: Collagen Vascular Diseases.

* Some patients had more than one diagnosis.

ϔ Warfarin, acetyl salicylic acid, low molecular weight heparin.

**Table-II:** The distribution of patients by months.

*Month*	*Number of cases*	*%*
January	23	9.9
February	17	7.3
March	29	12.5
April	23	9.9
May	37	15.9
June	18	7.8
July	12	5.2
August	18	7.8
September	15	6.5
October	13	5.6
November	17	7.3
December	10	4.3

**Fig.1:**
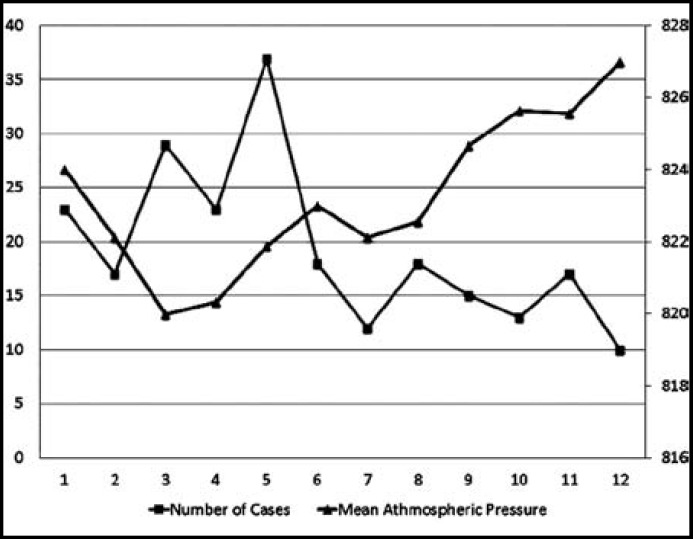
The relationship between atmospheric pressure with number of patients according to months. The relationship between the number of cases with atmospheric pressure, except January, was moving in the opposite direction. The most atmospheric pressure fluctuations was in January. Mean atmospheric pressure was 824 hPa (Minimum 818.4 hPa- maximum 829.9 hPa).

**Fig.2:**
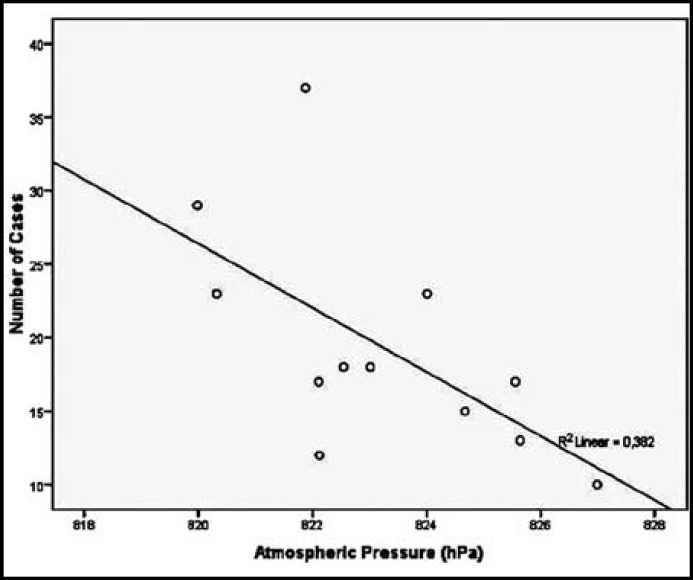
The relationship between atmospheric pressure and the number of cases

According to year, the numbers of patient admissions were 31 (13.4%), 29 (12.5%), 32 (13.8%), 43 (18.5%), 42 (21.1%), and 48 (20.7%) in 2006, 2007, 2008, 2009, 2010, and 2011, respectively. There were no difference between the years according to the atmospheric changes. The distribution of the cases according to month is shown in Table-II. The highest admission rates were in the spring season (in March, April, and May), the highest in May (n=37, 15.9%), and the lowest admission were in December (n=10, 4.3%). In January, the average atmospheric pressure 824.0 ± 3.25 (range, 813.5-829.9).Among the parameters related to atmospheric alterations (including mean atmospheric pressure, mean relative humidity, and mean outdoor temperature), a statistically significant negative correlation was found between the number of cases and mean atmospheric pressure (r=-0.618, p=0.032) (Fig.2) but not relative humidity (r=0.129, p=0.689) or outdoor temperature (r=-0.050, p=0.878). The distribution of the cases according to month and mean atmospheric pressure is shown in Fig.1. The number of case admissions was negatively correlated with the daily atmospheric pressure the day before (r=-0.633, p=0.027) and two days before (r=-0.603, p=0.038) but not on the day of hemoptysis (r=-0.569, p=0.054).

No significant relationship between diagnoses and their distribution according to month was found (p=0.841). Idiopathic cases were mostly admitted to the hospital in the spring months (40%) but showed no statistical significance (p=0.949). Additionally, 37% (n=27) of massive and 39% of submassive cases (n=62) were admitted in the spring months, but there was no statistical significance (p=0.762). 

## DISCUSSION

The findings show that more cases presented with hemoptysis in the spring months (March, April, and May, when atmospheric pressure is the lowest of the year) than in the other months. We could not determine any significant relationship between the increased number of cases and the other parameters, relative humidity and outdoor temperature.

Many studies indicate the association between seasonal atmospheric alterations and increased hospital admissions. Data from the US National Registry of Myocardial Infarction report approximately 53% more cases in the winter than during the summer, and the in-hospital case fatality rate shows a peak of 9% in the winter.^11^ Similarly, both non-reperfused and reperfused myocardial infarctions are smallest during the summer.^12^ Moreover, a winter peak for pulmonary thromboembolism,^13^^,^^14^ ischemic and hemorrhagic stroke,^15^^,^^16^ and rupture or dissection of aortic aneurysms,^17^ has been reported. In cardiovascular diseases, cold exposure, for example, may determine an increase in sympathetic activity and blood pressure levels, and a significant negative correlation between ambient temperature and blood pressure has been reported.^18^

The association of various lung diseases, such as pulmonary thromboembolism and pneumothorax, with seasonal atmospheric alterations has also been shown. For example, one study has reported a significant association between low atmospheric pressure and an increased incidence of pulmonary thromboembolism, and the incidence was increased especially in the spring season when atmospheric pressure is the lowest.^19^ In another study, in terms of the relationship between seasons and embolism cases, despite the determination of an insignificant positive correlation, a statistically significant positive correlation was determined between air pressure and humidity and case incidence.^20^ Previous studies have shown that hyperthrombocytopenia may occur at low atmospheric pressure.^21^ In cases with spontaneous pneumothorax, it has been shown that the atmospheric pressure one day and two days before patient admission was significantly lower and the maximum temperature on the day of admission was significantly lower compared to those without spontaneous pneumothorax.^22^ However, another study has shown that the seasons are not significantly related to the occurrence of spontaneous pneumothorax.^23^

More than one mechanism might be responsible for the development of hemoptysis. Bleeding is usually minimal when it arises from the pulmonary circulation (e.g., in the presence of left-sided cardiac disease); however, it can be substantial when it arises from the bronchial circulation because of the higher hydrostatic pressures promoting hemorrhage. Mechanical trauma, mostly secondary to forceful cough, can produce hemoptysis characterized by blood-streaked sputum; it tends to occur with an acute respiratory infection and is usually benign and self-limited. Foreign bodies can cause hemoptysis arising from bronchial circulation by direct injury to the bronchial wall. In patients with bronchiectasis, chronic bacterial colonization produces a change of the ciliated epithelial lining to a stratified squamous epithelium with areas of granulation tissue. Cough and recurrent infection damage the airway wall, while the supplying bronchial blood vessels become dilated and tortuous and develop extensive fragile bronchopulmonary anastomoses.^24^ Our study results suggest that low atmospheric pressure, especially in the spring season, might be another cause of hemoptysis and the increased number of cases due to presenting hemoptysis on those days. And also, there were no difference between the years according to the atmospheric pressure.

In some cases, the cause of hemoptysis cannot be determined despite thorough investigations.^8^ In the study of Bauley et al. examining the relationship between seasonal atmospheric alterations and idiopathic hemoptysis, they found that spontaneous idiopathic hemoptysis requiring hospitalization in France peaked in late winter and early spring (peak in March). In addition, hospitalizations for idiopathic hemoptysis followed a similar seasonal pattern, suggesting common triggering factors.^25^As idiopathic hemoptysis was increased in the spring season, we may speculate that it was the result of low atmospheric pressure as we found in our study, because Turkey and France are located in the same geographical climate zone and latitude. However, it is difficult to extrapolate these findings to regions located in the far north, the southern hemisphere, or the equator because of different climate conditions and seasonal variations. 

The number of cases in January was as high as that in the spring season, although there was no decrease in atmospheric pressure in that month. When we examined the meteorological data, we determined that there were more atmospheric fluctuations in January than in the other months. Thus, these fluctuations may be another reason for the increase in hemoptysis cases, although this hypothesis needs to be confirmed.

In summary, hemoptysis is very much influenced by weather factors; in particular, low atmospheric pressures significantly affect the development of hemoptysis. Fluctuations in atmospheric pressure may also play a role in hemoptysis. Patients at risk of hemoptysis should be closely followed up for relapse, especially in the spring season when there is an increased risk because of low atmospheric pressures. 

## Author Contribution:

Omer Araz, Metin Akgun, Mehmet Meral and Ali Metin Gorguner: Design of the study.

Elif Yilmazel Ucar and Yener Aydin: Data collection.

Leyla Saglam: Data analysis. Hasan Kaynar: Editing of the manuscript.

## Notes

***Source of funding:*** None declared.
